# Pea leafminer *Liriomyza huidobrensis* (Diptera: Agromyzidae) uses vibrational duets for efficient sexual communication

**DOI:** 10.1111/1744-7917.12598

**Published:** 2018-05-21

**Authors:** Jin Ge, Jia‐Ning Wei, Ding‐Jie Zhang, Chun Hu, De‐Zhi Zheng, Le Kang

**Affiliations:** ^1^ State Key Laboratory of Integrated Management of Pest Insects and Rodents, Institute of Zoology Chinese Academy of Sciences Beijing China; ^2^ University of Chinese Academy of Sciences Beijing China; ^3^ Key Laboratory of Zoological Systematics and Evolution, Institute of Zoology Chinese Academy of Sciences Beijing China; ^4^ School of Instrumentation Science & Optoelectronics Engineering Beihang University Beijing China; ^5^ Beijing Institutes of Life Science Chinese Academy of Sciences Beijing China

**Keywords:** biotremology, courtship behavior, *Liriomyza*, vibrational communication

## Abstract

The pea leafminer (*Liriomyza huidobrensis*) is a notorious pest of vegetables and ornamental plants worldwide. Despite a large number of studies on its biology and ecology, the courtship behavior and sexual communication of this species remain unclear. Here, we studied vibrational communication in the sexual interaction of the pea leafminer. On host plant leaves, females and males behaviorally displayed the bobbing‐quivering alternation, which finally led to copulation. Moreover, records of laser vibrometry revealed three‐signal duets underlying the behavioral alternation. Sexually mature males spontaneously emitted calls (MCs) to initiate the duets. The females rapidly responded to MCs by emitting replies (FRs) that are longer in duration. The FRs further triggered male replies (MRs) in their search for potential partners. Leafminer‐produced vibrational signals convey efficient information to partners and generate pair formation on stretched substrates, such as plant leaves and nylon mesh, but cannot elicit responses on dense substrates, such as glass and plastic. Vibrational playbacks of both MCs and FRs can elicit replies in females and males, respectively. This study completely characterizes substrate‐borne vibrational duets in a dipteran insect. The discovery of vibrational sex signals in the pea leafminer provides new insights for the development of novel approaches to control the pest and its relative species.

## Introduction

In most insect species, sexual communication at a long distance is thought to be mediated by air‐borne signals. For instance, female moths release sex pheromones to attract males (Roelofs *et al*., 2002), whereas male crickets produce songs to attract females (Zuk & Simmons, 1997). Regardless of the sex of the signaler, signal transmission in these two major sensory modes is unidirectional, and the sender–receiver role is fixed. Recent studies in biotremology have found that many hemipteran, plecopteran and neuropteran insects can exclusively use substrate‐borne vibrations for mate searching (Čokl & Virant‐Doberlet, 2003; Henry *et al*., 2013; Boumans & Johnsen, 2015). In these insect species, both sexes are likely to emit calling signals to initiate pair formation and partners often exchange signals in a stereotyped temporal pattern of duets, during which males locate the signaling females (Virant‐Doberlet *et al*., 2006; Čokl, 2008; Legendre *et al*., 2012; Rodríguez & Barbosa, 2014). Vibrational communication can largely facilitate sexual attraction and mate recognition in two aspects. First, it can propagate with minimal attenuation and overcome physical or chemical blockade imposed by foliage (Hill, 2009). Second, participation of both sexes in signaling reduces predation risk or increases search efficiency in the context of mate location, recognition and choice (Bailey, 2003; de Groot *et al*., 2011).

Most insects using vibrational sex communication are plant‐dwelling species (Čokl & Virant‐Doberlet, 2003). Some of them, including leafhoppers, stink bugs, psyllids, and whiteflies, are agricultural pests (Kanmiya, 2006b; Eben *et al*., 2015; Polajnar *et al*., 2016b; Nieri & Mazzoni, 2017). Thus, recent studies have proposed the use of substrate‐borne signals as a non‐chemical approach for communication interference (Eriksson *et al*., 2012; Polajnar *et al*., 2015). Moreover, playbacks of vibrational signals have already been proven effective for controlling hemipteran insects, both in the laboratory and in the field (Polajnar *et al*., 2016a).

As the second most diverse group of insects, Diptera has high economic importance, because many species are detrimental agricultural pests or vectors of diseases (Yeates & Wiegmann, 1999). Although the lack of volatile sex pheromones indicates the use of non‐chemical sexual communication in various species (Kanmiya, 2006a), the role of vibrational communication has long been underestimated in Diptera. Previous studies in the reed fly *Lipara* reported the presence of substrate‐borne acoustic signals of females, suggesting its role in eliciting searching behavior for males (Mook & Bruggemann, 1968; Kanmiya, 2006a). Recent studies have focused on *Drosophila melanogaster* and its two sibling species, in which the abdomens of males quiver to generate substrate‐borne signals and induce female immobility, thereby enhancing the receptivity of females in copulation (Fabre *et al*., 2012; Mazzoni *et al*., 2013). Although the roles of quivering signals in *Drosophila* are limited to close ranges where other modalities also play roles, these results indicate that substrate‐borne vibrations are involved in the sexual behavior of dipteran insects.

The *Liriomyza* leafmining flies (Diptera: Agromyzidae), the larvae of which mine plant leaves and stems for feeding, are pests of ornamental plants and vegetables worldwide (Spencer, 1973). Current methods based on insecticide sprays fail to effectively control *Liriomyza* pests because of their high insecticide resistance and high resurgence (Parrella *et al*., 1984). Thus, management of these pests requires development of new techniques to partially replace insecticide application. Trapping strategies or mating disruption by using vibrational playbacks can be applied for leafminer control if the vibrational communication of the insect species is uncovered. However, knowledge on courtship behavior and sexual communication in the leafminer species is limited, especially vibrational signals in sexual communication in *Liriomyza* remain elusive (Scheffer, 2000; Kang *et al*., 2009). To date, no sex pheromone in *Liriomyza* has been reported (Kang *et al*., 2009). An early observation has found a stereotypical mutual courtship on host leaves before copulation, and a female performs a bobbing motion and a male follows the female bobbing by body vibration, which possibly conveys substrate‐borne vibrational signals (Reitz & Trumble, 2002). Moreover, male vibrational signals during courtship have been recorded in a tentative experiment (Kanmiya, 2006a). Therefore, the sexual interaction in *Liriomyza* may involve vibrational communication.

In the present study, we aim to investigate the vibrational signals in the courtship and sexual communication of a polyphagous *Liriomyza* species, the pea leafminer (*Liriomyza huidobrensis*). To test the hypothesis that the pea leafminer relies on substrate‐borne vibrational duets for pair formation, we performed a series of experiments combining behavioral observation and laser vibrometry. Based on the analysis of the temporal structures of vibrational signals, we manipulated males or females to examine the correlation among signals, sexual maturity and pair formation. To test the effects of the substrate, we compared signal transmission and mating behavior on different substrates. Finally, by using vibrational playbacks, we attempted to verify if the recorded signals from males or females are sufficient to elicit replies from the opposite sex.

## Materials and methods

### Insects and plants

Pea leafminers were reared at 25 ± 2 °C and 60% relative humidity for 5 years (light : dark photoperiod: 14 : 10 h), with the kidney bean (*Phaseolus vulgaris* L. cv Naibai) as the host plant. Upon emergence, adults were isolated in microtubes individually and fed with 10% diluted honey for 2 days to reach sexual maturity or were immediately used.

Each bean plant was cultivated individually in plastic pots (8 cm in diameter) filled with a mixture of vermiculite and peat (1 : 2) in environmental chambers. Approximately 2‐week‐old plants with two fully developed true leaves were used in Mating on host plants and Substrate‐manipulated experiment sections. In Substrate‐manipulated experiment section, 1‐month‐old non‐host money plants (*Epipremnum aureum*) were used, the leaves of which were approximately the same size of those of bean plants. The money plants were cultivated following the rearing method for bean plants.

### Mating on host plants

All trials were conducted on kidney bean plants. Each plant was positioned within a circular opening (5 cm in diameter) of a custom‐made polymeric foam (15 × 15 × 3 cm), and the hole was covered with incised filter paper surrounding the stem. This set‐up created a platform and separated the upper and lower parts of the plant (Fig. S1A). The plant, together with the platform was placed into a cubic observation cage (15 cm × 15 cm × 20 cm) to prevent leafminers from escaping (Fig. S1A). A nylon mesh layer (400 mesh) was used to cover the cage on sidewalls and bottom, and a transparent membrane (5 *μ*m in thickness) was used as the roof.

A total of 55 pairs of leafminers were individually introduced into the arena. In each trial, the behaviors of males and females were recorded with two video cameras (HDR‐CX405, Sony, Tokyo, Japan) simultaneously at 25 frames per second from 9:00 hours to 16:00 hours on each experiment day. Each recording lasted for 900 s or until the end of copulation. Movies generated from one trial were imported to Observer 11.0 (Noldus Information Technology, Wageningen, Netherlands). They were then synchronized according to time marks in the video frame, and replayed on a computer. Each behavior displayed in the experiment was encoded using Observer software. The corresponding annotated data were assigned to the selected leafminer and recorded according to the time in an Observer file. Data processing was then repeated for the opposite sex, and the corresponding data were marked differently, although they were deposited in the same file according to their retention times. The decoded behavioral data were exported from Observer XT 11.0 to an Excel spreadsheet for further analysis. For the 41 pairs that displayed behavioral alternation between sexes, we monitored the numbers of alternations. The locomotion of the flies was difficult to measure because of their small (1–2 mm) body sizes and high mobility. Whenever possible, we assessed searching and mounting distances by using the auto‐tracking software Ethovision XT 8.5 (Noldus Information Technology, Wageningen, Netherlands). A 5 cm paper ruler was adhered on the leaves for tracking calibration.

Vibrational signals were registered from the bean leaves with a single‐point Doppler laser vibrometer (PDV‐100, Polytec, GmbH, Waldronn, Germany). Several pieces of reflective membrane were attached to the bean leaves to increase reflectance. The laser beam was directed to the reflective membrane close to the flies. Digital signals of vibration velocity were sampled with a USB digital audio interface (U24 XL; ESI Audiotechnik GmbH, Leonberg, Germany) using Adobe Audition CS6 software (Adobe Software, Seattle, WA, USA) at a sample rate of 48 kHz and a 16 bit resolution. Data were stored as .wav files in the computer. Analog signals were imported into the video camera to facilitate the correspondence of vibrational signals and behavior. Video and laser vibrometer recordings were synchronized during initialization by brief interruption of the laser path, by which both a momentary peak in the oscillogram and a black frame in the video were produced. In total, we sampled vibrational signals from five pairs of leafminers.

### Mating on nylon mesh

Nylon mesh was used as a substrate to characterize vibrational signals because it causes minimal distortion to signal frequencies and enhances signal amplitude (Elias & Mason, 2014). The arena was made by adhering the edge of a round piece of nylon mesh (6 cm in diameter) to the bottom half of a glass Petri dish (6 cm in diameter, Fig. S1B). Lights were directed from beneath to attract leafminers into the arena.

A total of 22 pairs were individually introduced into the arena. In each trial, the males and females were initially separated at a distance of approximately 3–5 cm. A video camera was positioned above the arena. Each recording lasted for 900 s or until the end of copulation. Vibrational signals were registered using the method described in Mating on host plants section.

### Male‐manipulated experiment

Male calls (MCs) generated by rapid flickering of a male body were initial signals that led to female replies (FRs) generated by a bobbing motion in duets. To test if MCs are specific to sexually mature males and essential for eliciting FRs, we introduced a 0‐day‐old male, 2‐day‐old male, or an immobilized 2‐day‐old male to a female 3–5 cm away on the nylon mesh arena (see Mating on nylon mesh section). Before introduction, males were observed for 1 min in the microtubes to determine whether they can exhibit the flickering behavior corresponding to MCs. Immobilized males were prepared by placing them in a tube on ice for 15 min. A video camera was used to record the occurrence of female bobbing behavior. Each recording lasted for 300 s.

### Female‐manipulated experiment

FRs led to male emission of replies accompanied with searching behavior. To test if FRs are specific to sexually mature females and essential for eliciting MRs and male searching, we introduced a 0‐day‐old female, a 2‐day‐old female, or an immobilized 2‐day‐old female to a male (following the method in Mating on nylon mesh section). Immobilized females were prepared by placing them in a tube on ice for 15 min. After introduction, a video camera recorded the occurrences of female bobbing, male quivering and searching for 300 s.

### Vision‐manipulated experiment

Vibrational motion can generate visual stimuli accompanied with vibrational signals (Gordon & Uetz, 2011). To exclude the possible roles of visual stimuli in duets, we observed pair formation in the dark (observed under red light) or in the light on the nylon mesh arena. In each trial, a pair of sexually mature leafminers was introduced on the nylon mesh arena. A video camera was used to record the occurrences of female bobbing, male quivering and searching for 300 s.

### Substrate‐manipulated experiment

Substrates can impose strong constraints on the transmission of substrate‐borne information (Elias & Mason, 2014). To test the effects of substrates in duets, we observed the premating behavior of pairs on five substrates: nylon mesh, plastic Petri dishes (6 cm in diameter), glass Petri dishes (6 cm in diameter) and two plant substrates, namely bean leaves and money plant leaves. For the leaf arenas, the leaves were dissected from the plants. The dissected leaves were maintained by placing their petioles into plastic Petri dishes filled with water and suspended in the air by adjusting the petioles to a right angle using parafilm (Fig. S1C). A pair of leafminers was introduced following the methods described in Mating on nylon mesh section and monitored for 300 s on each of the five substrates. The occurrences of female bobbing, male quivering and searching were recorded.

We assessed the effect of substrate on vibrational properties of MCs. A male was subsequently introduced randomly to the five substrates and 5 cm away from the laser monitoring point. Each record of vibrational signals lasted for 1 min. We measured and averaged three characteristics of the two elements in MCs: pulse duration, dominant frequency and relative amplitudes.

### Playback experiment

A playback experiment was conducted to assess if substrate‐borne vibrations alone can elicit a reply in adult leafminers. A headset loudspeaker with the central area made of membrane was used (3.7 cm in diameter, 32 Ω, K550, Edifier, Beijing, China). The loudspeaker was controlled by the sound card mentioned earler using Adobe Audition software from a computer. Vibrational signals were registered using the abovementioned method to monitor the playbacks and responses (see Mating on host plants section). Moreover, a video camera was used to record the duetting behaviors, including female bobbing, male quivering and searching. The arena was placed on sand to reduce noise interference. To prevent escape from the loudspeaker arena, the wings of 1‐day‐old flies were partially removed at the distal end (approximately 1/3 of wing area was removed) with microscissors under a stereo microscope. Except for the inability to fly, wing‐amputated flies normally walked and fed as untreated flies did.

The amplitudes of playback signals were adjusted to the level of naturally emitted signals recorded on a nylon mesh. In the playback experiment to stimulate females, we randomly selected MCs emitted by one male from our library of nylon mesh recordings. The stimulatory sequence was 1.59 s long and consisted of four MCs (Audio S1, pulse repetition rate = twice per second; pulse duration of element A = 26 ± 4 ms; pulse duration of element B = 44 ± 1 ms). The stimulatory sequence was repeated thrice and separated at an interval of 10 s. Females were scored as responsive if they emitted at least one FR. In the playback experiment to stimulate males, we randomly selected FRs emitted by one female from our library of nylon mesh recordings. The stimulatory sequence was 3.27 s long and consisted of five FRs (Audio S2, pulse repetition rate = 1.61 times per second; pulse duration = 117 ± 5 ms). The stimulatory sequence was repeated three times and applied once the tested males produced MCs. Males were scored as responsive if they emitted at least one MR.

### Statistics and terminology

Most behaviors observed in Mating on host plant section were defined according to the previous description on the mating behavior of *L. huidobrensis* (Reitz & Trumble, 2002; Table S1). “Quivering” was used to describe male courtship behavior according to a previous study on *Drosophila* (Fabre *et al*., 2012). The ethogram of premating behavior was established by sequential analysis with a single‐order Markov chain. The analysis of the matrix was performed using a collection of public domain Java applets for behavioral data (available at http://caspar.bgsu.edu\~software\java). The significance of the transition was assessed by cellwise examination (*P <* 0.05) (Chen *et al*., 2002). The correlation between the behavior duration and occurrence of copulation was assessed by a binary logistic regression, in which a forward stepwise approach was performed.

We define “elements” as separated subunits composing a “signal”. Each element is defined as a “pulse” if it is a homogenous parcel of sound with a finite duration (Broughton, 1963). The stored .wav files were imported as external data into Obersver XT 11.0 together with their corresponding films to identify waveforms generated from each behavior. After noise filtering by using the noise reduction module of Adobe Audition, the .wav files were imported into MatLab R2013b (MathWorks, Natick, MA, USA). The spectral properties of the oscillogram were analyzed by Fast Fourier Transformation type Hamming, with a window length of 2048 samples and 99% overlap. The following parameters were measured: pulse duration, pulse repetition rate (times of pulses per second), and dominant frequency. Data are presented in the Results section as means and standard deviations, together with the numbers of leafminers (*N*) from which signals were obtained and the numbers of signals (*n*) analyzed.

Different percentages of flies exhibiting a certain behavior were compared by two‐sided Fisher's exact test according to the analysis of nominal behavioral data in a previous study (Dweck *et al*., 2015). The parameters of vibrational signals were first checked for normality with Shapiro–Wilk normality test. The MC repetition rates before and after FRs were compared by nonparametric Wilcoxon matched pair test. The vibrational characteristics in the Substrate‐manipulated experiment section were accordingly analyzed either with nonparametric Kruskal–Wallis test or parametric repeated analysis of variance (ANOVA) followed by Tukey's multiple comparison. All data were processed and analyzed using SPSS 17.0 (SPSS Inc., Chicago, IL, USA).

## Results

### Female bobbing and male quivering alternate on plants

**Figure 1:**
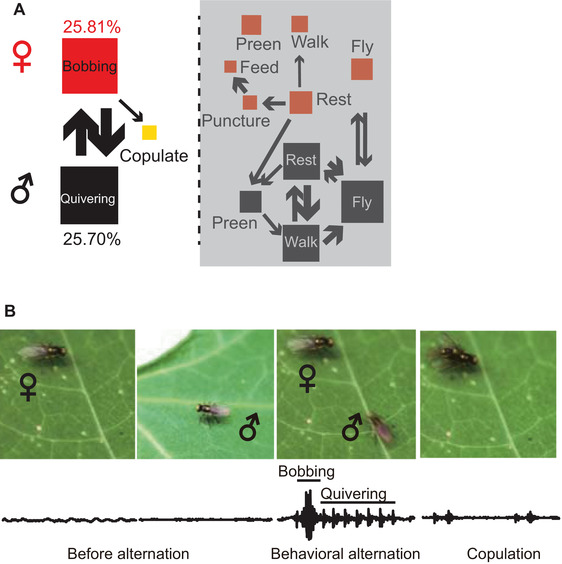
Premating behavior and vibrational signals on bean leaves. (A) Ethogram of premating behavior constructed based on the first‐order transition of behaviors. The size of the boxes representing each behavior is proportional to the relative frequency of occurrence of a particular behavioral pattern. The color of the boxes represents female (red) or male (black) behavioral elements. Behavioral transitions are depicted as arrows when they occur significantly more often than predicted by chance. The arrow size indicates the degree of possibility in transition. (B) Frames of a video and a vibrational oscillogram of vibration of behavior before, during and after a behavioral duet. Each clip of vibrational signals is shown in a 1 s time frame.

Nine behavioral parameters of pea leafminers before copulation were recorded, and three of them were identified as sex‐specific (Table S1). The ethogram demonstrated that mating behavior involves female bobbing, male quivering and their copulation. Female bobbing and male quivering account for 25.81% and 25.70% of the total counts of all behavioral parameters, respectively. Copulation is led by the bobbing‐quivering alternation but is independent of the other behaviors (Fig. 1A). This correlation was further supported with a binary logistic regression model, indicating significant correlation between the duration of this alternation and copulation occurrence (Table S2). Among the 55 pairs tested, 41 pairs exhibited bobbing–quivering behavioral alternation between sexes, whereas the other 14 pairs did not show any interaction. In a stereotypical sexual interaction (Movie S1), a female produced conspicuous bobbing in a fixed position. Following female bobbing, males responded by quivering their bodies, and this behavior preceded female bobbing in the next repetition of alternation. During the bobbing–quivering alternation, males searched for their potential female partners (3.66 ± 1.33 cm, *N* = 11 pairs). After approximately 28 alternations (28 ± 13, *N* = 41 pairs), the male found the female and attempted a mount from a distance of 0.40 ± 0.16 cm (*N* = 17 pairs). A total of 34 females accepted mounting males by staying motionless, whereas seven females rejected copulation by kicking the males or running away. Laser vibrometry of the leaves revealed vibrational signals corresponding to the behavioral alternation. Specifically, female bobbing generated a single pulse, whereas male quivering generated 3–7 pulses (*N* = 5 pairs, Fig. 1B). No exchange of signals was observed in copulating pairs, although a two‐element signal was recorded during copulation.

### Vibrational duets are composed of three signals

**Figure 2:**
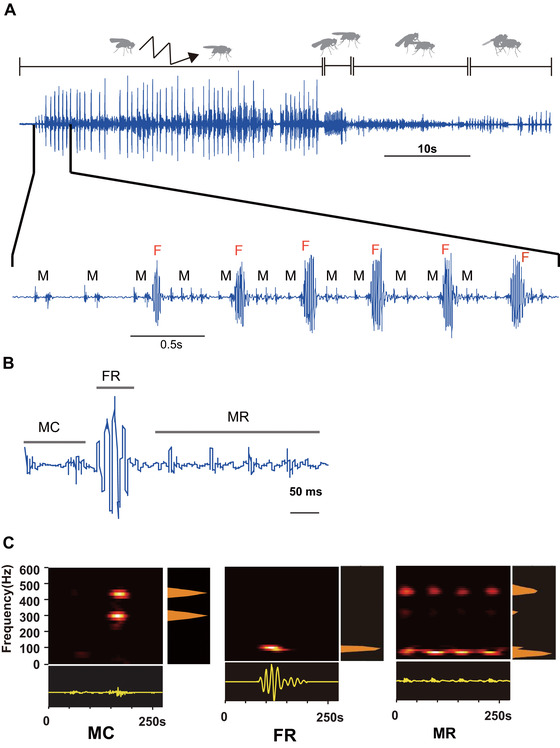
Mating behavior and three‐signal duets on nylon mesh. (A) Oscillograms of mating behavior of paired adult leafminers recorded on a nylon mesh. Six repetitions are shown below at higher resolution. M: pulses produced by males. F: pulses produced by females. (B) A representative duet. Male calls (MC) generated by male body flickering: male call, female response (FR) generated by female bobbing: female reply, male response (MR) generated by male quivering: male reply. (C) Oscillogram (at bottom), spectrogram (at top left), and instantaneous amplitude spectrum (at right) of each signal in a duet.

Pairs of adult leafminers demonstrated premating behavior on the nylon mesh arena similar to that on bean leaves. (Fig. 2A, Movie S2). By using a nylon mesh as substrate, the laser vibrometry recorded a double‐element pulse preceding each pulse generated by female bobbing (Fig. 2B). This signal was generated by a slight and quick male body flickering (Movie S3). Irrespective that only 14 pairs in 22 trials exhibited bobbing–quivering alternation between sexes, all males could spontaneously emit these initial signals. These male calls (MCs) were repeated 3.37 ± 1.13 times per second, consisting of two elements separated at an interval of 61.3 ± 17.8 ms on average (Fig. 2A and 2B). Element A lasted for 24.2 ± 4.7 ms on average at a dominant frequency of 327 ± 101 Hz. Element B lasted for 42.3 ± 7.1 ms on average at a dominant frequency of 305 ± 89 Hz (*N* = 14, *n* = 40, Fig. 2B and 2C and Table 1). After 2–5 repetitions (3.4 ± 0.9, *N* = 14) of MCs, a female bobbing replies (FRs) almost immediately after offset of the last MCs (latency of the FR: 1.7 ± 1.4 ms, *N* = 14, *n* = 70, Fig. 2B). An FR is a single pulse signal lasting for 112 ms on average with a dominant frequency of 78 Hz (*N* = 14, *n* = 140, Fig. 2B and 2C and Table 1). Thirty‐one milliseconds later, the male began to quiver and produced replies (MRs) with 2–7 pulses repeated at the rate of 14 times per second (*N* = 14, *n* = 248, Fig. 2A and Fig. 2B). Each pulse in MRs lasted 71 ms and had a harmonic structure with dominant frequency of 66 Hz (*N* = 14, *n* = 248, Fig. 2C). During MRs, the male moved 4.7 ± 1.8 mm, which is approximately three times the male body size (*N* = 14, *n* = 74). At 35 ms after the last pulse of MRs, the male stopped and emitted an MC again (35 ± 18, *N* = 14, *n* = 74). The repetition rate of MCs after FRs decreased to 1.65 ± 0.32 (*N* = 14, *n* = 140) compared with MCs repetition rate before FRs (Wilcoxon matched pairs test, *N* = 14, *P* = 0.01).

**Table 1:** Vibrational characteristics of components in duets of *Liriomyza huidobrensis* recorded on nylon mesh

Signals		Pulse repetition rates (pulses/s)	Number of elements		Pulse duration (ms)	Dominant frequency (Hz)
Female	FR	–	1		111.63 ± 19.42	77.79 ± 12.20
Male	MC	3.37 ± 1.13	2	Element A	24.2 ± 4.7	327 ± 101
				Element B	42.3 ± 7.1	305 ± 89
	MR	14.44 ± 0.70	1		71 ± 12	231.38 ± 58.75

FR, female response; MC, male call; MR, male response.

### MCs and FRs are essential for replies in sexual communication

**Figure 3:**
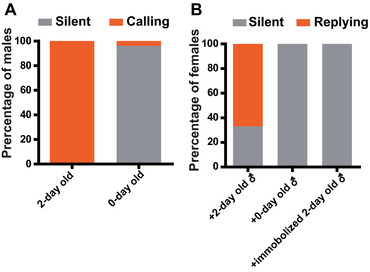
Male‐manipulated experiment. (A) Percentages of signaling males in the 2‐day‐old or 0‐day‐old groups. (B) Percentages of signaling females paired with 2‐day‐old, 0‐day‐old, or immobilized 2‐day‐old males.

In the male‐manipulated experiment, all 2‐day‐old males spontaneously produced MCs. However, only 4% of 0‐day‐old males produced MCs (*N* = 30 and 23 for 2‐day‐old and 0‐day‐old males, respectively. Fisher's exact test, *P* < 0.0001, Fig. 3A). The 2‐day‐old males induced 67% of females to emit FRs. By comparison, neither 0‐day‐old males nor immobilized 2‐day‐old males triggered any FR in females **(** *N* = 20 for immobilized 2‐day‐old males, Fisher's exact test: *P* < 0.0001; Fig. 3B).

**Figure 4:**
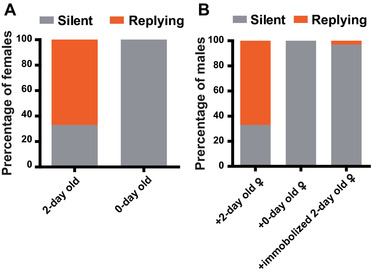
Female‐manipulated experiment. (A) Percentages of signaling females in 2‐day‐old or 0‐day‐old groups. (B) Percentages of signaling males paired with 2‐day‐old, 0‐day‐old, or immobilized 2‐day‐old females.

In the female‐manipulated experiment, 67% of the 2‐day‐old females emitted FRs. This proportion is extremely different from the absence of FRs in the 0‐day‐old female group (*N* = 27 and 21 for the 2‐day‐old and 0‐day‐old groups, respectively; Fisher's exact test, *P* < 0.0001, Fig. 4A). Zero‐day‐old or immobilized 2‐day‐old females elicited 0% and 2.9% of males to reply, respectively. However, 2‐day‐old females elicited 67% of males to reply (*N* = 34 for immobilized 2‐day‐old females, Fisher's exact test: *P* < 0.0001; Fig. 4B). Specifically, in trials where females emitted FRs, 94% of males (17 of 18) were responsive. All the responsive males located the signaling females and attempted to mount.

### Duets are substrate‐dependent

All males emitted MCs under dark and light conditions and elicited approximately 60% of females to produce FRs (*N* = 20 and 19 in the light and dark conditions, respectively; Fisher's exact test, *P* = 0.75, Fig. S2). Replying females induced MRs in all males under both dark and light conditions.

**Figure 5:**
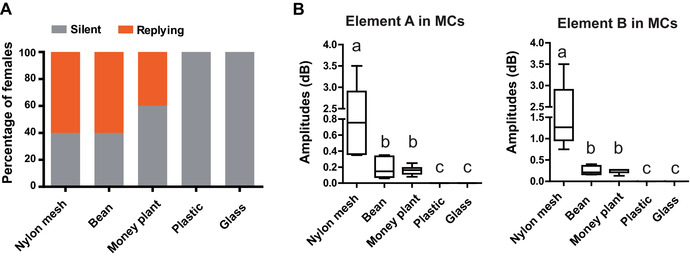
Substrate‐manipulated experiment. (A) Percentages of signaling females paired with males on five substrates. (B) Measured amplitudes of two elements of male calls (MCs) on five substrates. Different letters show significant differences between amplitudes after Kruskal–Wallis test followed by Dunn's multiple comparison.

In the substrate‐manipulated experiment, all males emitted MCs on five substrates. The percentages of females emitting FRs were not different in the nylon mesh, bean leaves, and non‐host money plant leaves (*N* = 20, 20 and 23 for nylon mesh, bean leaves, and money plant leaves, respectively; Fisher's exact test: *P*_nylon mesh versus bean leaves_ = 1.0, *P*_nylon mesh versus money plant leaves_ = 0.37, Fig. 5A). On these three substrates, FRs exclusively elicited MRs in males, which then located the replying females. However, on plastic and glass, none of the females replied to the males and none of the males emitted MRs (*N* = 20 for both treatments; Fisher's exact test: *P*_nylon mesh versus plastic or glass_ < 0.0001). The five substrates filtered MCs in various manners. On plastic and glass, MCs were not detected. The measured relative amplitudes were high on nylon mesh and low on bean and money plant leaves (*N* = 6 for each treatment, Kruskal–Wallis test followed by Dunn's multiple comparison test, for element A: Kruskal–Wallis statistics = 11.08, *P* = 0.0039; for element B: Kruskal–Wallis statistics = 11.46, *P* = 0.0032, Fig. 5B). Moreover, the dominant frequency and pulse duration were similar on these three substrates (*N* = 6 for each treatment, repeated ANOVA followed by Tukey's multiple comparison, for element A: pulse duration, *F* = 1.66, *P* = 0.24, dominant frequency: *F* = 0.30, *P* = 0.74; for element B: pulse duration, *F* = 0.05, *P* = 0.95, dominant frequency: *F* = 0.16, *P* = 0.85, Fig. S3).

### Vibrational playbacks elicit behavioral responses in duets

**Figure 6:**
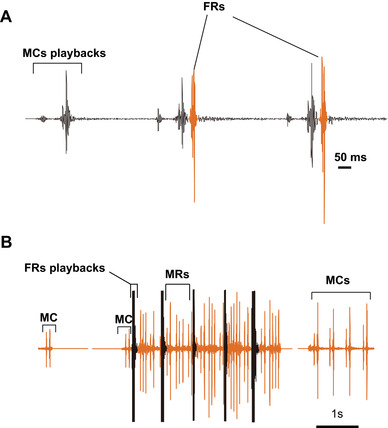
Playback experiment. (A) Oscillogram of female responses (FRs) of a responsive female replying to male call (MC) playbacks. (B) Oscillogram of male responses (MRs) of a responsive male replying to FR playbacks.

On the loudspeaker membrane, 60% of females were responsive to playbacks of MCs (*N* = 15, Fig. 6A). Similar to those stimulated by natural MCs, FRs stimulated by MC playbacks immediately followed the offset of recorded MCs (latency of the FR: 1.76 ± 1.08 ms, *N* = 15, *n* = 25, Fig. 6A, Movie 4). Ninety‐five percent of males were responsive to the playback of FRs (*N* = 22). All responsive males performed a searching behavior (Movie 5). Upon the FR offset, males stopped producing MRs and only emitted MCs (*N* = 21, Fig. 6B).

## Discussion

The present study revealed a substrate‐borne duetting system, in which vibrational signals play important roles in the pair formation in dipteran insect species. The general pattern of duets in *L. huidobrensis* involves three steps as follows. First, males initiate duets by spontaneously emitting calls (MCs). Second, receptive stationary females respond by producing replies (FRs); and third, males emit replies (MRs) to FRs and track the signaling females. The identification and playback of vibrational signals in the duets found a reliable female stimulus to attract males, thus offering a potential way for the development of a new strategy to trap the insect species. In addition, the role of MCs and FRs in pair formation provides insights into the mate recognition and mate location in plant‐dwelling dipteran insects.

MCs are emitted by sexually mature males even in the absence of females, and are thus spontaneous signals in the duets of pea leafminers. We showed that the repetition rates of MCs can be adapted to the occurrence of female signals. After FRs, the repetition rates decreased by more than half. Similarly, many male insects in duetting can modulate calling rates (Batemen, 2001) or the intensity of signals (van Staaden & Römer, 1997; Helversen *et al*., 2001) once a female is attracted. Such plasticity in male advertisement signals may reflect male strategic investment in mate searching under the selective pressure of eavesdropping or energetic burden (Bailey, 2003).

FRs in duets are vital in male mate searching in the pea leafminer because males failed to locate silent females in the experimental set‐ups. This result is consistent with previous studies that males in several species rely on female vibrations transmitted through substrates for orientation and directionality (Mazzoni *et al*., 2014; Gibson & Cocroft, 2018). Remarkably, in the pea leafminer, FRs are twice as long as MCs. In addition, the temporal structure of FRs elicited by natural MCs and MC playbacks revealed a small distance (approximately 0.002 s) between MCs and FRs compared with those in other species (Mazzoni *et al*., 2009b; Rodríguez & Barbosa, 2014; Nieri & Mazzoni, 2017). For small plant‐dwelling insects, locating partners is challenging (Virant‐Doberlet *et al*., 2006). The smaller body size of male leafminers may incur difficulty in orienting mates. The robust female reply can provide reliable information about the location, thereby reducing male costs in mate searching. In addition, given that copulation stimulates egg‐laying (Parrella, 1987) and brings female seminal gifts (Kaspi & Parrella, 2008) in *Liriomyza*, a rapid male localization can not only provide females a priority to lay their eggs on host plants but also enhance the chance of receiving nuptial donations. Therefore, females may also benefit from FRs, especially when males are scarce in the environment.

In response to FRs, males searched for females and produced vibrational replies (MRs), which are time‐locked to FRs and higher in repetition rate than MCs. In their repeated numbers of pulses, MRs have a high degree of variation and thus are likely to convey information for further female assessment of potential mates. In addition, the MR long pulse duration is likely to result in evolution of alternative mating tactics. Previous studies have found that in leafhoppers and treehoppers, males can produce competitive masking signals to interfere with other males’ pair formations (Derlink *et al*., 2018; Nieri *et al*., 2017), or silently approach duetting females by stealth (Mazzoni *et al*., 2009; Legendre *et al*., 2012; Kuhelj *et al*., 2016). Whether the variance in MRs leads to bias in female preference and whether male pea leafminers exhibit alternative mating tactics require further investigation.

The substrate choice in mating suggests the evolution of sex signals to match the characteristics of the environment. Although plant species may influence substrate‐borne communication because of varying efficiencies of transmission associated with the plant physical properties (Cocroft *et al*., 2006), no difference was observed in the MC signal parameter between host leaves and non‐host leaves and similar percentages of pair formation were found in the two substrates. The results indicated possible sexual interaction on non‐host plants at a closer range. However, on plastic and glass, males and females did not establish communication through vibration. Further studies should examine the signal transmission at a long range to estimate the full‐scale effect of plants on the vibrational communication of the pea leafminer.

The mechanism of vibrational signal production in the pea leafminer remains elusive. Although an early morphological investigation has suggested a stridulatory apparatus consisting of a haired file on the basal tergites and a ridged scraper on the inner part of the hind femur (Tschirnhaus, 1971), a recent study found no contact between the femur and file during sound production (Kanmiya, 2006a). Regardless of the mechanism, the duetting signals depend on soft substrates to transmit because MCs are severely attenuated and fail to elicit FRs and a proper pair formation on dense substrates (such as plastic and glass). This result is consistent with the effect of substrates on the mating of parasitoids (Joyce *et al*., 2008) and jumping spiders (Elias *et al*., 2004), the seismic courtship signals of which are also steeply attenuated on dense substrates (glass and plastic for parasitoids, rock and sand for jumping spiders).

The playback vibrations of MCs and FRs were sufficient to elicit a reply in males and females, respectively. Males seemed nondiscriminatory to female FRs, because FRs elicit almost all males to exhibit searching and to reply with MRs. Therefore, FR is a crucial stimulus to attract males. A recent work conducted on the stink bug *Halyomorpha halys* has proven that the exploitation of female vibrational signals is effective to manipulate male behavior (Mazzoni *et al*., 2017). The discovery of FRs in the pea leafminer provides a theoretical basis to design specific acoustic traps, which are harmless to the environment and non‐target organisms, especially under the context that sex pheromones have not been reported in *Liriomyza* to date (Kang *et al*., 2009). It would also be feasible to integrate the female vibrational signals into currently existing integrated pest management strategies including colored sticky traps (Kang, 1996) and push‐pull methods using plant attractants or repellents (Kang *et al*., 2009).

In summary, in the pea leafminer, males and females exchange substrate‐borne vibrational signals to establish pair bonding before copulation. MCs initiate the duets and FRs induce male searching behavior. The success of vibrational playbacks in triggering specific behavioral reactions suggests the potential applications of the signals in developing acoustic trapping strategies. Whether other *Liriomyza* species can use substrate‐borne duets for sexual communication remains an open question. Further studies should also focus on the signaling space of vibrations in laboratory and field trapping experiments.

## Disclosure

The authors declare no conflict of interest.

## Supporting information


**Fig. S1**. Arenas used in experiments. (A) Arena in mating on host plant; (B) Nylon mesh arena; (C) leaf arena.

**Fig. S2**. Percentages of signaling females paired with males under light or dark conditions.

**Fig. S3**. Measured dominant frequency and pulse duration of two elements of MCs on different substrates.

**Table S1**. Definition and sexual dimorphism of behaviors of *Liriomyza huidobrensis*.
**Table S2**. Variables retained in binary logistic model to fit copulation occurrence and parameters of other behaviors.

**Audio S1**. Stimulatory sequence of the male calls (MCs) in playback experiment.

**Audio S2**. Stimulatory sequence of the female responses (FR)s in playback experiment.

**Movie S1**. Behavioral alternation of female bobbing and male quivering before copulation on bean leaves.

**Movie S2**. Behaviors and the corresponding audio signals (analog signals from laser vibrometer) in duets on a nylon mesh.

**Movie S3**. Male body flickering defined as male calls (MCs). The wings were amputated for clarity.

**Movie S4**. Female emission of female responses (FRs) responding to male call (MC) playbacks. Audio signals are analog signals from a laser vibrometer.

**Movie S5**. Male emission of male responses (MRs) and searching behavior responding to female response (FR) playbacks. Audio signals are analog signals from a laser vibrometer.
