# Supplementary material for: Pea leafminer Liriomyza huidobrensis (Diptera: Agromyzidae) uses vibrational duets for efficient sexual communication
Source: Insect Sci. 2018 May 21;26(3):510–22. doi: 10.1111/1744-7917.12598 (PMC7379950; doi:10.1111/1744-7917.12598)
Supplement: Supplementary file 4 — Table S1. Definition and sexual dimorphism of behaviors of Liriomyza huidobrensis. Table S2. Variables retained in binary logistic model to fit copulation occurrence and parameters of other behaviors. [file INS-26-510-s001.doc]

## Supporting information


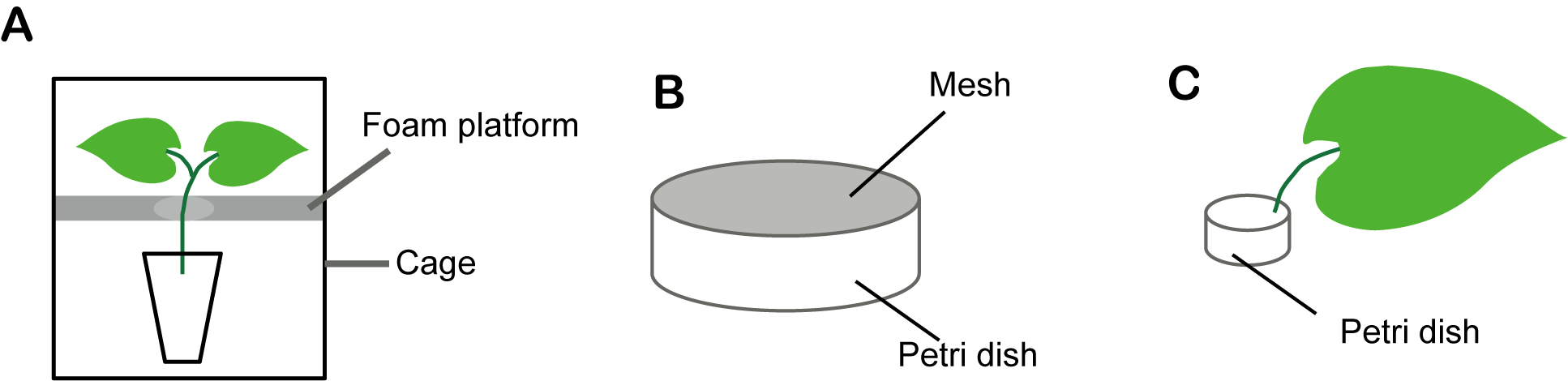


**Figure S1** Arenas used in experiments. (A) Arena in mating on host plant; (B) Nylon mesh arena; (C) Leaf arena.

**
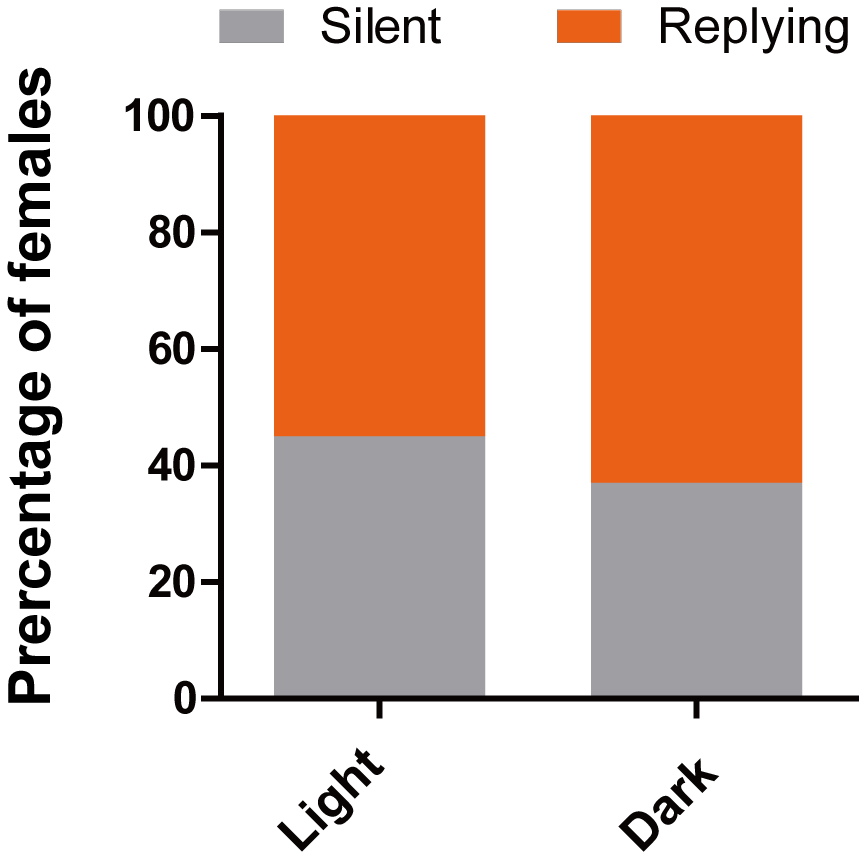
**

**Figure S2** Percentages of signaling females paired with males under light or dark conditions.


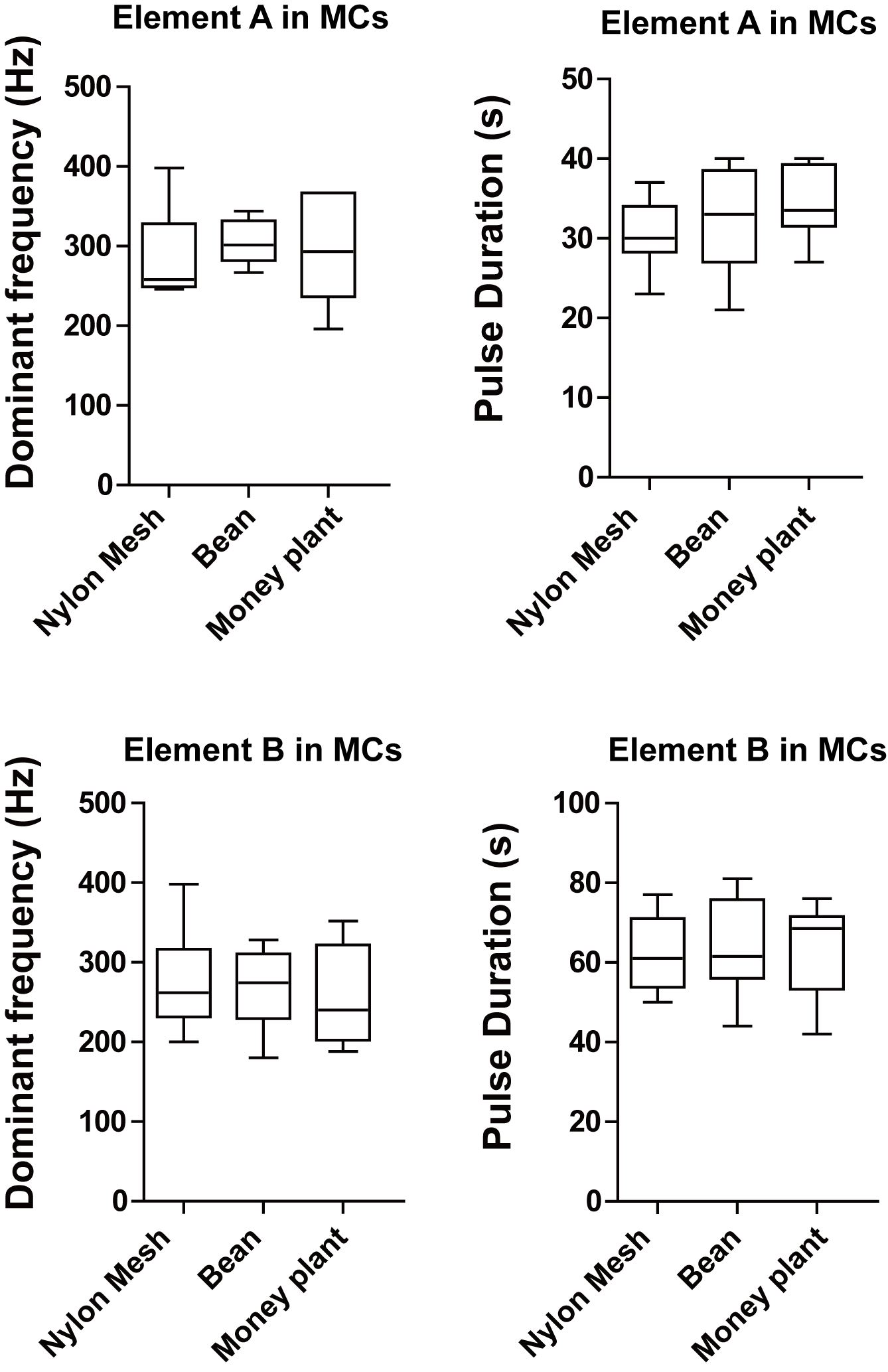


**Figure S3** Measured dominant frequency and pulse duration of two elements of MCs on different substrates.

| **Table S1.** **Definition and sexual dimorphism of behaviors of *L. huidobrensis*** | | | |
| --- | --- | --- | --- |
| Behaviors | definition | Females | Males |
| Walk | Change position by gentle direct moves on the substrates | + | + |
| Rest | Hold the position | + | + |
| Fly | Change position by gentle direct flights in the air | + | + |
| Feed | Sponge food using its mouth part | + | + |
| Preen | Brush the surface of body by legs | + | + |
| Bobbing | vigorous body up and down motion | + | - |
| Puncture | Puncture wholes by ovipositor | + | - |
| Quivering | Move towards partners with rapid adjustment of direction and with body trembling | - | + |
| Copulate | Males mount females and insert the aedeagus into the reproductive tract of the female | + | + |

| **Table S2 Variables retained in binary logistic model to fit copulation occurrence and parameters of other behaviors** | | | | | | |  |
| --- | --- | --- | --- | --- | --- | --- | --- |
| Variables in the Equation | β | Standard error | Wald | df | Sig. | Exp(B) |  |
| Female feed duration | 7.634 | 10.718 | .507 | 1 | .476 | 2067.264 |  |
| Male feed duration | -30.321 | 47848.775 | 4.016E-07 | 1 | .999 | 6.788E-14 |  |
| Female puncture rates | -2.729E-01 | 4.580 | .004 | 1 | .952 | .761 |  |
| Male walk duration | -2.801 | 3.889 | .519 | 1 | .471 | .061 |  |
| Male rest duration | -1.776 | 3.893 | .208 | 1 | .648 | .169 |  |
| Female walk duration | .383 | 4.532 | .007 | 1 | .933 | 1.467 |  |
| Female rest duration | 1.938 | 4.035 | .231 | 1 | .631 | 6.942 |  |
| Male preen duration | .909 | 2.982 | .093 | 1 | .761 | 2.481 |  |
| Female preen duration | 1.404 | 4.368 | .103 | 1 | .748 | 4.071 |  |
| Male fly rates | .254 | .273 | .864 | 1 | .353 | 1.289 |  |
| Female fly rates | -1.257E-01 | .961 | .017 | 1 | .896 | .882 |  |
| Bobbing-quivering duration | 16.143 | 5.811 | 7.717 | 1 | .005 | 1.025E+07 |  |
| Constant | -7.525E-01 | 2.976 | .064 | 1 | .800 | .471 |  |

**Audio S1** Stimulatory sequence of the MCs in playback experiment.

**Audio S2** Stimulatory sequence of the FRs in playback experiment.

**Movie S1** Behavioral alternation of female bobbing and male quivering before copulation on bean leaves.

**Movie S2** Behaviors and the corresponding audio signals (analog signals from laser vibrometer) in duets on a nylon mesh.

**Movie S3** Male body flickering defined as MCs. The wings were amputated for clarity.

**Movie S4** Female emission of FRs responding to MC playbacks. Audio signals are analog signals from a laser vibrometer.

**Movie S5** Male emission of MRs and searching behavior responding to FR playbacks. Audio signals are analog signals from a laser vibrometer
